# Chaotic optical power dropouts driven by low frequency bias forcing in a mid-infrared quantum cascade laser

**DOI:** 10.1038/s41598-019-40861-7

**Published:** 2019-03-14

**Authors:** Olivier Spitz, Jiagui Wu, Mathieu Carras, Chee-Wei Wong, Frédéric Grillot

**Affiliations:** 1 LTCI Télécom ParisTech, Université Paris-Saclay, 46 rue Barrault, Paris, 75013 France; 2 mirSense, Centre d’intégration NanoInnov, 8 avenue de la Vauve, Palaiseau, 91120 France; 3 Fang Lu Mesoscopic Optics and Quantum Electronics Laboratory, University of California Los Angeles, Los Angeles, CA 90095 USA; 4 College of Electronic and Information Engineering, Southwest University, Chongqing, 400715 China; 5 Center for High Technology Materials, University of New-Mexico, 1313 Goddard SE, Albuquerque, NM 87106 USA

## Abstract

Mid-infrared quantum cascade lasers operating under external optical feedback and external periodic bias forcing are shown to exhibit a deterministic chaotic pattern composed of frequencies which are linked to the one of the forcing. Results also show that both the amplitude and the frequency of the forcing play a key role in the number of retrieved spikes per modulation period. These findings are of paramount importance for chaotic operation of quantum cascade lasers in applications such as optical countermeasure systems and secure atmospheric transmission lines, as well as for simulating neuronal systems and the communication between neurons due to sudden bursts.

## Introduction

Quantum cascade lasers (QCLs), which have found increasing application since their first experimental demonstration in 1994^1^, are unipolar semiconductor lasers based on intersubband transitions within the conduction band. Mid-infrared QCLs can operate in single or multimode configuration, in continuous wave or pulsed operation^2^. They were first operated at cryogenic temperatures where efficient heat dissipation eases the implementation of continuous wave operation and, later, at room temperature with thermo-electrical cooling for real-world application requiring versatility^3^. Such application applications include, for instance, optical countermeasures, light detection and ranging (LIDAR), remote sensing, gas spectroscopy or free-space communications^4^. The mid-infrared spectrum is composed of two atmospheric windows which are highly relevant for communication purposes^5^ and GHz modulation experiments at room temperature have recently been reported to assess the possibility of long-range and high-speed data transmission using mid-infrared QCLs^6^. The emission properties of QCLs can be controlled by reinjecting part of the output back into the laser, using external optical feedback. This technique showed relevant modifications on the emission and dynamical properties of the QCLs^7^. External optical feedback has a strong influence on the QCL dynamics and several feedback regimes have already been identified with optical spectrum analysis^8^. Experiments with lasers pumped with a quasi-continuous wave showed that QCLs exhibit a chaotic behavior with low frequency fluctuations (LFF) both at room temperature^9^ and cryogenic temperature^10^. These experimental investigations also showed that the sensitivity to external feedback was strongly dependent on temperature owing to a modification of the upper state lifetime of the laser, which was confirmed by numerical simulations^11^. These experiments were the proof of concept of temporal chaos in such lasers because QCLs are theoretically known to be more resistant to external optical perturbations. Indeed, they have a small linewidth enhancement factor and a carrier-to-photon lifetime ratio around 0.1^12^, well below that of interband diode lasers which can reach up to 10000. This high value explains why diode lasers are good candidates when it comes to study chaotic patterns in semiconductor lasers under external optical feedback^13^. As the LFF dynamics is highly excitable, prior works have reported on the impact of current modulation and periodic forcing on the chaotic patterns. That can be meaningful for manipulating the phase-space dynamics with a view toward developing more secure communication lines. For instance, several numerical^14,15^ and experimental^16–20^ works widely describe the entrainment phenomenon when applying an external optical feedback to a semiconductor laser while periodically modulating the laser current. However, most of them focus on modulation frequencies of several dozens or hundreds of MHz in order to have a forcing frequency close to the external cavity frequency. Consequently, a limited number of papers deal with low modulation frequencies below 5 MHz^21–25^. Entrainment phenomenon corresponds to a shift of the frequency of an oscillator in order to synchronize to that of a periodic forcing. Such work has been described in electrical, mechanical and biological systems^26^. Indeed, in the latter, triggered spikes are the main means of communication between neurons in the neuronal network^27–29^ and semiconductor lasers operating under the LFF regime and a periodic forcing are relevant candidates to mimic neuronal activities. When describing the entrainment phenomenon in semiconductor lasers, one of the key parameters is the *q*:*p* criterion^21^ where *q* represents the number of dropouts occurring in the time-series every *p* periods of the forcing. This criterion profoundly varies when modifying the frequency of the periodic forcing, but no main discrepancies were reported when varying the low amplitude of the forcing in laser diodes^24^. This work investigates for the first time the synchronized chaotic fluctuation-induced optical bursts in a mid-infrared QCL and more precisely, the entrainment phenomenon. We also investigate the influence of a sine forcing at a few MHz over the power dropouts which are typical features of the LFF dynamics. The QCL is pumped with a continuous bias, above threshold in order to remain in optimal conditions for chaos observation and a periodic forcing with an amplitude between 8% and 50% of the continuous wave is applied. Overall, this experimental study is of paramount importance for controlling and understanding the dynamics of intersubband semiconductor lasers. The long-term purpose of our study being to implement secure atmospheric transmission lines and disruptive technologies such as unpredictable optical countermeasure systems with mid-infrared QCLs.

## Results

**Figure 1:**
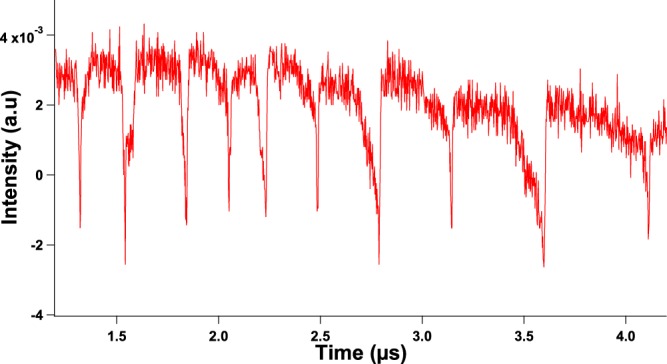
Experimental time trace of a LFF chaotic pattern in the QCL under study pumped with a quasi-continuous bias at 430 mA in a cryogenic environment (77 K). The time interval between successive dropoffs is not constant, which is a typical feature of LFF dynamics.

**Figure 2:**
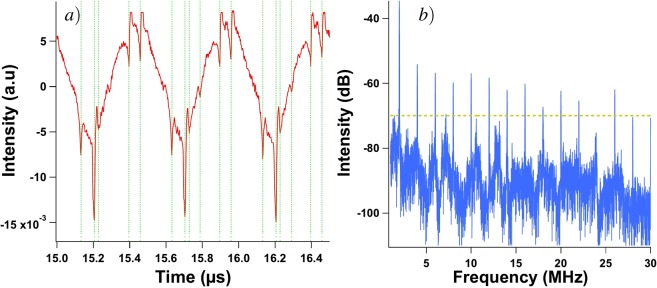
Experimental time trace close-up (**a**) and RF spectrum (**b**) of the QCL’s emitted wave when a periodic forcing at 2 MHz is applied with external optical feedback; green vertical lines represent the dropout occurrences in the time trace and same figure without these lines can be found in Fig. 4(c); the interval between two spikes in the RF spectrum is exactly the modulation frequency *f*_*m*_ = 2 MHz and the dashed line represents the intensity threshold taken into account for Figs 5–7.

**Figure 3:**
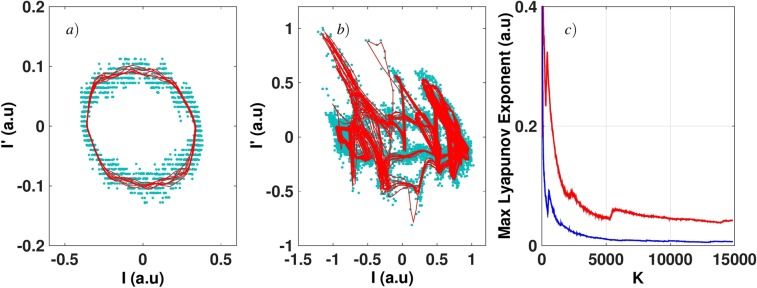
Dynamics analysis of the experimental waveforms. Phase portrait corresponding (**a**) to Fig. 4(a,b) to Fig. 4(b). Blue dots are the measured raw data and the solid red curves represent the noise-reduced orbital trajectories. (**c**) gives the calculated largest Lyapunov exponent (LLE) related to Fig. 4(a) (blue curve) and to Fig. 4(b) (red curve).

**Figure 4:**
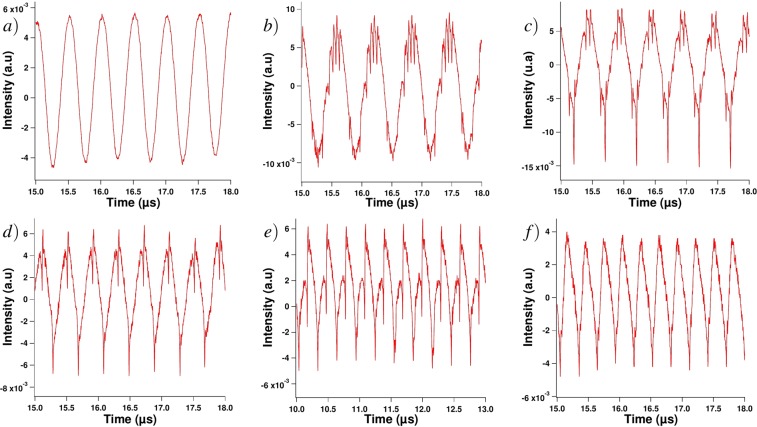
Experimental time traces when external optical feedback is applied to the QCL and with a sine forcing of the continuous wave of: 1.6 MHz (**b**), 2 MHz (**c**), 2.7 MHz (**d**), 3.3 MHz (**e**), 3.4 MHz (**f**); trace (**a**) corresponds to the case when no external optical feedback is applied and the modulation frequency is 2 MHz. All the traces were retrieved for a continuous bias of 430 mA and a modulation amplitude of 120 mA.

If a QCL is pumped far from threshold with a continuous wave or a quasi-continuous wave and a strong enough external optical feedback applied, the laser shows chaotic behaviors with LFF patterns. This non-linear phenomenon is characterized by fast oscillations related to the external cavity (with frequencies around 400 MHz in our case^10^) and slow oscillations with frequencies of several MHz related to the dropouts of the LFF^9^. Fig. 1 shows an example of a chaotic pattern driven by LFF dynamics for a quasi-continuous wave of 430 mA applied to the QCL under study. The frequency of the quasi-continuous bias is 200 kHz with a duty cycle of 90%, which means that the pulses are 4.5 *μ*s long with an interval of 0.5 *μ*s between them. In this case, the dropouts do not exhibit a periodic behavior because the time interval between these dropouts varies. This result is similar to what was already reported when studying the main feedback regimes and the bifurcation process from steady state to LFF dynamics through a limit cycle in QCLs^9,10^. The LFF can be understood as a competition between modes and a stability process around the mode with highest gain. The elliptic trajectory wanders around an external cavity mode for a few revolutions. It then hops to another mode with a higher intensity, repeating the process until it reaches the highest order mode where the collision with its antimode produces the dropoff^30^. When, in a second step, periodic forcing is added, the time interval between LFF spikes seems to remain constant, as can be seen in Fig. 2 when a continuous bias of 430 mA and a peak-to-peak sine forcing of 120 mA at 2 MHz are applied. To confirm the chaotic behavior of the waveforms with external optical feedback and external periodic forcing, we carried out a dynamical analysis through the Lyapunov exponents (LEs) from the experimental time traces. LEs describe the divergence rate of nearby attractor trajectories and these criteria have been widely used to characterize chaos in nonlinear systems such as semiconductor lasers^31^. Figure 3 shows the phase diagram corresponding to Fig. 4(a,b) and reveals an attractor behavior in the case where both external optical feedback and external periodic forcing are applied. Furthermore, Fig. 3(c) emphasizes that the largest LE converges towards a strictly positive value when the external optical feedback is applied, contrary to what happens without this feedback.

**Figure 5:**
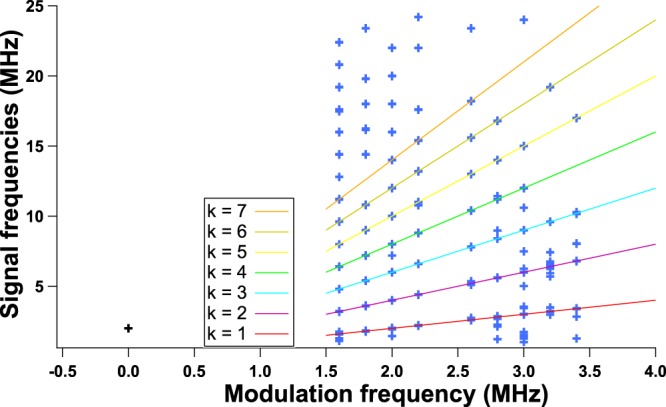
RF spectrum of the chaotic signal when applying a 120 mA peak-to-peak sine periodic forcing to the QCL under a continuous bias of 430 mA; blue markers correspond to the RF spectrum frequencies above −70 dB, when external optical feedback is applied for modulation frequencies shown on the x axis while the black marker represents the RF spectrum of the signal when a 2 MHz modulation is applied without external optical feedback; solid lines are for visual guidance for the reader and represent integral multiples of the modulation frequency: *f*_*s*_ = *k* × *f*_*m*_ with *k* displayed on the diagram.

**Figure 6:**
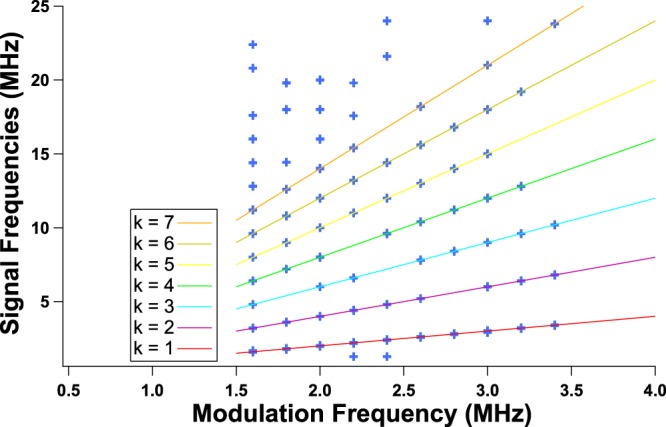
RF spectrum of the chaotic signal when applying a 120 mA peak-to-peak sine periodic forcing to the QCL under a continuous bias of 350 mA; blue markers correspond to the RF spectrum frequencies above −70 dB, when external optical feedback is applied for modulation frequencies shown on the x axis; solid lines are for visual guidance for the reader and represent integral multiples of the modulation frequency: *f*_*s*_ = *k* × *f*_*m*_ with *k* displayed on the diagram.

**Figure 7:**
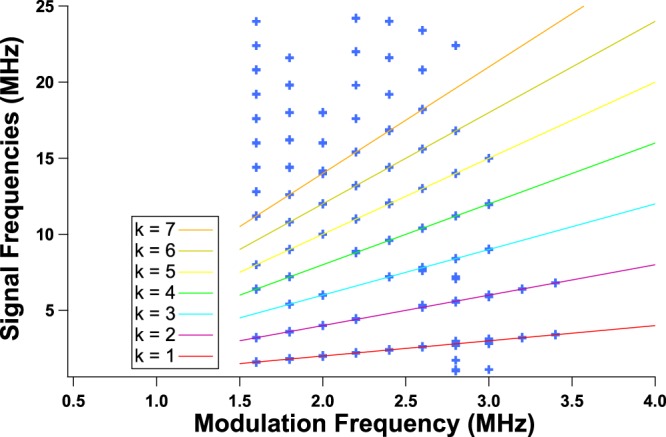
RF spectrum of the chaotic signal when applying a 120 mA peak-to-peak sine periodic forcing to the QCL under a continuous bias of 530 mA; blue markers correspond to the RF spectrum frequencies above −70 dB, when external optical feedback is applied for modulation frequencies shown on the x axis; solid lines are for visual guidance for the reader and represent integral multiples of the modulation frequency: *f*_*s*_ = *k* × *f*_*m*_ with *k* displayed on the diagram.

**Figure 8:**
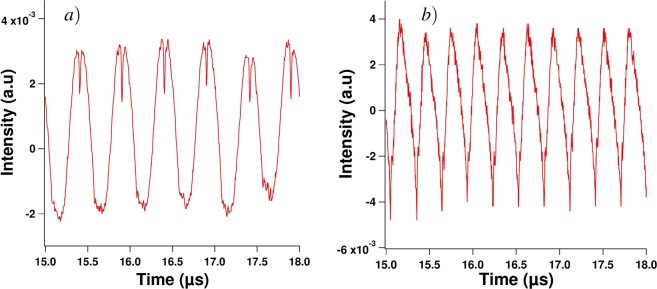
Experimental time traces for a continuous bias of 350 mA and a modulation amplitude of 40 mA (**a**) and for a continuous bias of 430 mA and a modulation amplitude of 120 mA (**b**); both experimental waveforms illustrate the condition *q* equals 1.

While the time interval between dropoffs is not necessarily constant and takes values which are uncorrelated to the dynamical system in the case of a continuous bias^32–35^, applying a periodic electrical forcing fosters specific frequencies. As can be seen in Figs 5–7 from the RF spectrum analysis for various continuous biases, these frequencies are mostly integral multiples of the modulation frequency, even though this relationship seems to slowly vanish when increasing the modulation frequency above 3 MHz. This may be due to the cutoff frequency of the current source which is optimized for a sine modulation up to 3 MHz. Figure 5 gathers all the frequencies that can be found in the RF spectrum analysis of the QCL under external optical feedback and periodic forcing when the frequency of this periodic forcing takes values between 1.6 and 3.4 MHz in steps of 0.2 MHz. Each marker represents a frequency with a retrieved intensity above −70 dB in the case where the QCL is pumped with a continuous bias of 430 mA and a modulation amplitude of 120 mA. Most of the markers appear on one of the solid lines representing integer multiples of the modulation frequency, which means that the time interval between successive LFF spikes strongly depends on the forcing frequency. The same conclusion can be drawn for Figs 6 and 7. Even if the RF spectrum seems to globally show the same behavior whatever the modulation frequency, the time traces are quite different in terms of the distribution of the spikes, as shown in Fig. 4. This behavior can be studied using the aforementioned *q*:*p* criterion. In our case, the frequency of the spikes is always higher than or equal to the frequency of the forcing, so we set *p* to 1. Tables 1 and 2 gather the *q* values for several continuous biases and modulation amplitudes when the frequency of the forcing is 1.5 MHz and 2 MHz. These tables show that, on the one hand, *q* slowly varies when increasing the value of the continuous bias. On the other hand, *q* depends on the variations of the amplitude, which is not the case of standard laser diodes^18^. Not only the frequency of the periodic forcing, but also the amplitude of the periodic forcing has a preponderant influence on the time interval between spikes. Figure 4 shows that the *q* parameter deeply varies for the range of frequencies under study. For instance, *q* equals 1 for a modulation frequency of 3.4 MHz, and 6 for a modulation frequency of 2 MHz when the QCL is pumped with a 430 mA bias and when the amplitude of the modulation is 120 mA. From 1.5 to 2.7 MHz, each period of the sine wave gathers multiple LFF spikes and it is difficult to foresee a specific organization of the LFF dropoffs. When the modulation frequency is above 2.7 MHz, only two LFF patterns occur per wave period and the dropoffs always occur for a fixed phase of the periodic forcing, which is when the extrema of the sine wave are reached. Eventually, for frequencies above 3.4 MHz, the frequency of the spikes is exactly the same as the forcing frequency (Fig. 4(f)) or, in other words, *q* equals 1. However, as mentioned in the section describing the experimental setup, the low-noise current source has a cutoff frequency around 3 MHz and even if the sine modulation is still visible in the time traces with no external optical feedback, the amplitude of the forcing is less important compared to the modulations with frequency below 3 MHz. It is worth noticing that in the case where the period of the dropoffs and the period of the sine forcing are the same, or *q* equals 1, the spikes appear for a given phase, as previously described. Nevertheless, the value of this phase can vary for two different configurations. For instance, Fig. 8 shows that the phase shift is *π* even though the value of *q* remains 1. Further investigation is needed to determine what can make spikes occur simultaneously with maxima or minima of the sine forcing.

**Table 1:** *q* value with related uncertainties when the frequency of the sine forcing is 1.5 MHz.

Continuous bias	350 mA	430 mA	530 mA
Modulation amplitude			
40 mA	3	4	4
80 mA	7 ± 1	8 ± 1	8 ± 1
120 mA	10 ± 2	10 ± 1	12 ± 1
160 mA	14 ± 2	14 ± 2	15 ± 1

**Table 2:** *q* value with related uncertainties when the frequency of the sine forcing is 2 MHz; the value with the dagger corresponds to the time trace and RF spectrum of Fig. 2 and the value with the asterisk corresponds to the time trace of Fig. 8(a).

Continuous bias	350 mA	430 mA	530 mA
Modulation amplitude			
40 mA	1*	2	2
80 mA	3	5	5 ± 1
120 mA	6 ± 1	6 ± 1^†^	8 ± 1
160 mA	8 ± 1	9 ± 1	9 ± 1

## Discussion

We experimentally reported on the first observation of chaos synchronization induced optical bursts in a mid-infrared QCL. Using external optical feedback and periodic sine forcing of several MHz, the QCL operating in the LFF regime displays frequencies which are integer multiples of the forcing frequency. This differs from what was observed with only external optical feedback. In such case, the chaotic pattern exhibits time intervals whose frequency can not be related to one of the parameters of the system. This means that the distribution of the dropoffs is significantly modified by the modulation of the current bias. We furthermore found that the spikes observed in the waveforms related to the LFF occur for a given phase shift. This phase shift depends on the amplitude of the periodic forcing, the value of the continuous bias applied to the QCL and the frequency of the sine forcing. Further investigation will determine the influence of the shape of the modulation (square signal, asymmetric triangular pulses or sine forcing) as well as the evolution of the *q*:*p* ratio when increasing the modulation frequency. The external cavity frequency is actually set to be of a few hundreds of MHz. Experimental studies with laser diodes which achieved to match the frequency of the forcing with the frequency of the external cavity showed the absence of LFF dropouts for a narrow range of modulations^17^. It is also relevant to notice that the studied range of modulation frequencies did not allow us to exhibit ghost frequencies^36^ in the RF spectrum analysis. Indeed, all the system frequencies, which include the frequencies of the LFF spikes, were at least equal to that of the forcing. It also appears that the dynamics of the system is more affected by variations in the modulation amplitude than by changes in the continuous pumping bias. We thus derived that the two parameters of prime importance in order to control the number of spikes per period are the frequency and the amplitude of the modulation while the value of the continuous bias is kept constant. One particular source of applications of such experimental results concerns the field of optical communication systems and optics-based information security. Indeed, various encoding schemes, with or without modulation of the pump, have been reported^37,38^, with the need of a receiver laser driven by a transmitter laser. The chaotic pattern produced by the master laser is injected into the slave laser, with similar characteristics. The slave laser consequently becomes chaotic and reproduces the chaos of the master laser. This dynamic between the slave and the master laser can be combined with message encryption methods in order to transmit a secure message from the transmitter to the receiver^39^. In order to make it difficult for observers to detect the transmitted symbols, modulation can be considered to further tailor the phase-space dynamics. Indeed, encoding the signal by modulating the pump across the maxima of intensity could be a much better method to improve the privacy of the communication channel compared to low-dimensional chaotic communication processes for which reconstructing the system’s chaotic attractor is enough to decipher the encoded message^40,41^. In other words, using the interspike interval with periodic modulation between dropout events allows spanning the phase space and constitutes a useful partition for chaotic communications provided that the modulation frequency is not too large (otherwise it becomes difficult to monitor the spikes’ locations). Last but not least, these findings are also of importance to better understand neuronal activities and the communication between neurons due to sudden bursts as well as other physiological processes relying on non-linear phenomena^42,43^.

## Methods

**Figure 9:**
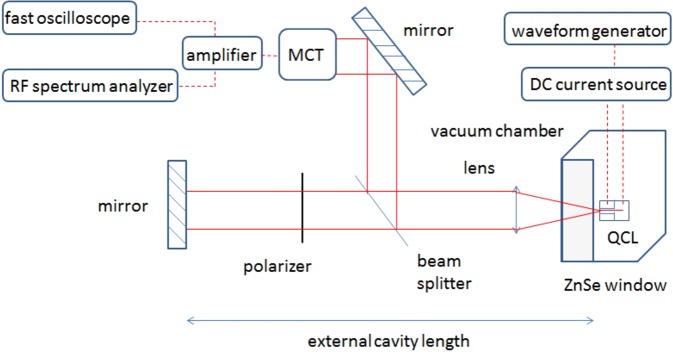
Experimental setup with the feedback path allowing control over the back-reflected light and the detection path.

**Figure 10:**
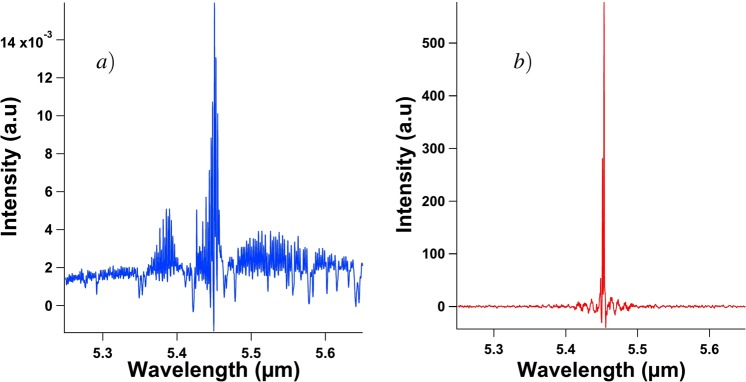
Spectral characteristics of the free-running QCL operating at 77 K and under a continuous bias just below threshold at 325 mA (**a**) and above threshold at 340 mA (**b**).

**Figure 11:**
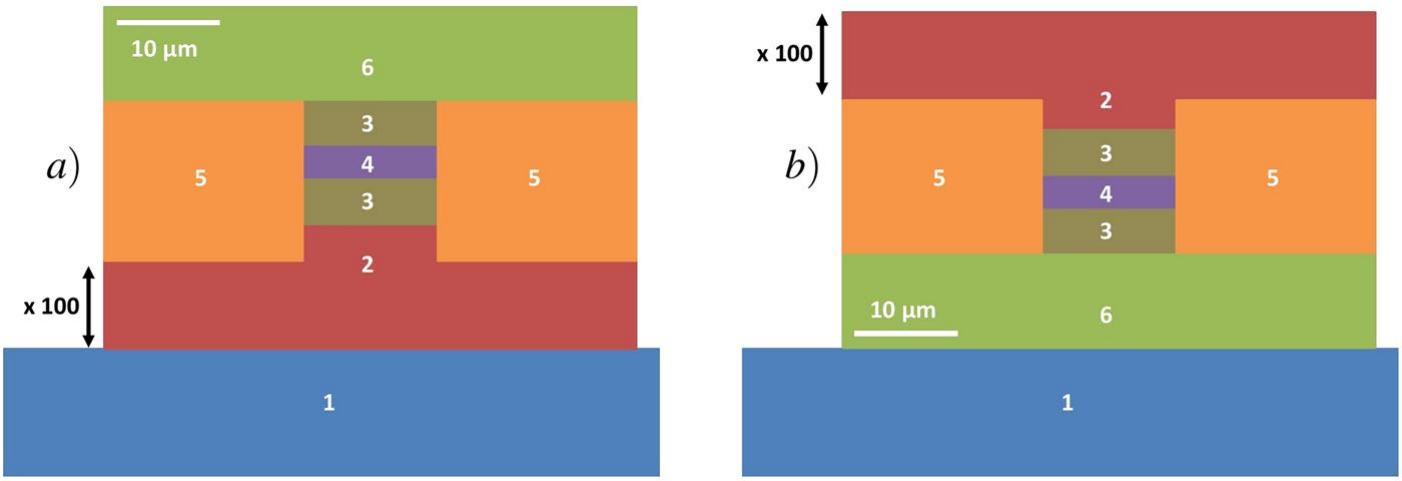
Schematic of the emission facet of a QCL which is epi-side up mounted (**a**) and epi-side down mounted with mounting base (1), substrate (2), cladding (3), active region (4), dielectric region (5) and gold metalization (6). The substrate is one hundred times wider than shown on the schematic and the mounting base can be a few millimeters wide and several centimeters large.

The experimental set-up used to carry out the measurements at the temperature of boiling nitrogen is shown in Fig. 9 and is similar to the one described in ref.^10^. The length of the external cavity is set to a value of 27 centimeters. Experiments are implemented at a cryogenic temperature of 77 K, allowing the laser to be pumped with a continuous wave. The current source in this experiment is a low-noise source (Wavelength Electronics QCL2000 LAB) and the continuous bias delivered by the source can be modulated with an external signal from a waveform generator (Rigol DG1022Z). In this experiment, we use a sine forcing with a frequency varying between 1.5 MHz and 3.5 MHz. It is relevant to notice that the low noise source has a modulation bandwidth of 3 MHz and that the measurements carried above this value lead to a forcing with an amplitude that is smaller than that expected because of the low-pass filter embedded in the current source. To analyze the data, both a real-time oscilloscope at one giga sample per second (Atten ADS112CAL) and a RF spectrum analyzer (Agilent Technologies CXA N9000A) are linked to the high bandwidth room-temperature mid-infrared detector (Vigo PEM Mercury-Cadmium-Telluride; MCT). The signal retrieved from this detector is magnified with a low noise amplifier (RF BAY, Inc LNA-545) with 500 MHz bandwidth, in order to overcome the background noise. The QCL we investigated is 2 mm long and 14 *μ*m wide. Figure 10(a) shows that, below threshold, this QCL behaves as a multimode laser. When biased above threshold, it emits a single mode at a 5.45 *μ*m wavelength as can be seen in Fig. 10(b). The device is a distributed feedback (DFB) laser from mirSense with a metal grating cladding at the top of the laser ridge. To allow the light to be emitted from the QCL but also the back-reflected wave to couple inside the laser cavity, one of the facets of the laser is highly reflective and the other one is cleaved to have a 70% transmission coefficient. The laser ridge is made of two InP cladding layers surrounding the 1.5 *μ*m active region, which consists of thirty stacks of AlInAs/GaInAs grown with a molecular beam epitaxy technique. The width of this QCL is inspired by a design from ref.^44^. The whole electronic sandwich is gold-tin welded to an AlN substrate to ensure good thermal dissipation, which is furthermore optimized by the epi-side down configuration (detailed in Fig. 11(b)) of the device. Indeed, the QCL is to be powered with a modulated continuous wave and this induces a strong warm-up of the whole structure. The configuration of Fig. 11(b) is more thermally efficient than that of Fig. 11(a) because the active region of the QCL is one hundred times closer to the mounting base linked to the temperature heat sink. This laser has a threshold current of 331 mA at 77 K and a maximum emitted power for a bias current of 950 mA. In the results presented above, the laser is pumped at a continuous bias of either 350 mA, 430 mA or 530 mA and a sine forcing between 40 mA and 160 mA, with steps of 40 mA, is applied. For instance, if the laser is pumped with a continuous wave of 430 mA and if the amplitude of the applied forcing is 120 mA, which corresponds to the widely described case of Fig. 4, then the laser is pumped between 430 mA and 550 mA. Furthermore, the amplitude of the periodic modulation is of paramount importance because if the amplitude is too low, the influence of the forcing is not seen and the chaotic behavior is similar to what is retrieved with a pure continuous bias. When the amplitude of the modulation is too high, the RF spectrum analysis gives similar results but the amplitude of the chaotic pattern becomes small compared to that of the forcing and the LFF dynamics cannot be analyzed as precisely with the time traces. This would hence lead to huge uncertainties in the *q* value. The external optical feedback applied to the QCL is the same as the maximum value applied in ref.^10^ when a cryostat is implemented, which corresponds to a feedback ratio of nearly 9%. Thus, it is important to stress that self-mixing effects cannot be involved in this work because the feedback mirror is motionless and the feedback ratio is greatly above the regular feedback ratio required for self-mixing interferometry^45^.

## Data Availability

The data that support the plots within this paper and other findings of this study are available from the corresponding authors upon reasonable request.
